# Physiological effect and pharmacokinetic evaluation of combined oral administration of cannabidiolic acid and cannabigerolic acid in dogs

**DOI:** 10.3389/fvets.2026.1779760

**Published:** 2026-02-25

**Authors:** Monique M. Koll, Robert Menardi, Daniel Hughes, Wayne S. Schwark, Joseph J. Wakshlag

**Affiliations:** 1 Quicksilver Integrative Veterinary Sports Medicine and Rehabilitation, Spring, TX, United States; 2 ElleVet Sciences Inc., South Portland, OR, United States; 3 Department of Clinical Sciences, Cornell University, Ithaca, NY, United States

**Keywords:** cannabichromenic acid, cannabidiolic acid (CBDA), cannabigerolic acid (CBGA), dog, HEMP, pharmacokinetics

## Abstract

**Introduction:**

The study of hemp-based cannabinoids has focused primarily on cannabidiol (CBD). *Cannabis sativa* however, naturally synthesizes cannabidiolic acid (CBDA) as opposed to the better-known neutral derivative. The precursor to cannabidiolic CBDA is cannabigerolic acid (CBGA) and cultivar extracts that contain both acidic cannabinoids are available as animal supplements. Pharmacokinetically these acidic cannabinoids are often superior to the neutral cannabinoids. The combinations of cannabinoids may affect their bioavailability and has not been well studied. Pharmacokinetics and safety of 90 day administration of a combination of CBGA and CBDA were examined in dogs.

**Methods:**

Eight adult beagles between 10 and 11 kg were provided a whole hemp blended extract containing primarily CBDA (1 mg/kg) and CBGA (1 mg/kg). Initial single dose 24-h pharmacokinetics were performed, and from day 2–90 dogs were dosed similarly at 7 a.m. and 3 p.m. daily and serum concentrations relative to steady state were examined at days 27, 57 and 90. Complete blood counts, serum chemistry and physical examination were performed at these time points to examine physiological effects.

**Results:**

Both CBDA and CBGA were detected in the bloodstream with AUC geometric means of 1,383 ng-h/mL and 930 ng-h/mL, respectively, on 24 h pharmacokinetics assessment. Both minor cannabinoids cannabichromenic acid (CBCA) and tetrahydrocannabinolic acid (THCA) were also observed throughout the trial, while neutral cannabinoids such as cannabidiol (CBD) could not be found. Monthly cannabinoid analysis revealed both CBDA and CBGA in the range of 10–175 ng/mL 6 h after morning dosing for all dogs. THCA and CBCA were observed in the serum of dogs at significant concentrations in the 5–75 ng/mL range. All physical exam findings and blood work were unremarkable.

**Conclusion:**

An equal blend of primarily CBDA and CBGA whole hemp acidic cannabinoid rich oil in soft gel appears to be well tolerated by dogs over a 90 day period with no adverse events. Pharmacokinetics are like other CBDA and CBGA studies in dogs with surprising concentrations of the other minor cannabinoids THCA and CBCA. Considering the favorable absorption/retention profile of the acidic cannabinoids, further research for therapeutic interventions for inflammatory conditions and organ dysfunction should be considered.

## 1 Introduction

Phytocannabinoids, from the *Cannabis sativa* plant, have seen an increase of their use in both the human and veterinary medical fields in the past several years. Many studies show they have value for medical and behavioral conditions such as pain, epilepsy and anxiety in both humans and dogs (1–7).

This increase in popularity is a result of the 2018 farm bill which allowed for the federally legal growth and distribution of hemp, opening the doors to cannabinoid-based nutraceutical products with less than 0.3% THC. The primary phytocannabinoid of interest has been cannabidiol (CBD). The hemp plant, however, synthesizes cannabidiolic acid (CBDA) from the parent cannabinoid known as cannabigeriolic acid (CBGA) (8). These acids are the native forms found in the plant. Only under the high heat of extraction and/or concentrating processes will they be decarboxylated into CBD or cannabigerol (CBG) (9). The acidic cannabinoids are variable across hemp cultivars. The most prominent acidic cannabinoids found are often CBDA, CBGA, tetrahydrocannabinolic acid (THCA), and cannabichromenic acid (CBCA) with a range of other less abundant derivatives depending on hydroxylation, hydration or alkyl tail length (8). These cannabinoid acids can be extracted intact using cold hydrocarbon-based extraction methods and likely have similar biological activity to the decarboxylated neutral cannabinoids, all without the psychotropic activity of delta 9 tetrahydrocannabinol (THC) (10).

In 24-h pharmacokinetic studies in dogs and many other species, both CBDA and CBGA have been shown to have a superior pharmacokinetic profile when compared to their neutral counterparts (11–15).Though rarely studied clinically, this superiority makes them favorable targets for clinical investigation.

The acidic compounds have been previously postulated to be unstable as isolates which has discouraged the pharmaceutical industry from pursuing them clinically. They appear, however, to be relatively stable when isolated and formed into whole hemp oil tinctures utilized to make products (16, 17).

CBD and CBG are two of the most widely studied therapeutic compounds, and are often isolated from *Cannabis sativa* extracts. When administered orally, they have less than 20% bioavailability in dogs because of first-pass effect of liver metabolism, which can be influenced by form (oil, capsules etc) and the effect of administration with food (18). Canine liver microsome studies have shown important differences in the metabolism of CBDA versus CBD. The acidic cannabinoids appear to be directly glucuronidated and may not undergo phase 1 hydroxylation and carboxylation before conjugation (19). These differences could be part of the reason for differences in absorption and retention in 24–72 hrpharmacokinetic studies.

CBDA has been utilized in a mixed cannabinoid product examining safety in dogs over a 90 day period and has been used therapeutically in a product for mobility, seizures and atopic dermatitis successfully (2, 16, 20, 21). CBGA is in its infancy regarding demonstrated clinical utility, but has been shown to have organ protecting and profound antioxidant effects like CBDA (22–25). However, CBGA administration has not been examined in dogs for beyond 2 weeks of administration as part of a CBG and CBGA mixed product (26). Considering the increased bioavailability, COX inhibition, and superior antioxidant potential of the acidic cannabinoids, it would be ideal to examine their clinical therapeutic efficacy (27–29).

There are short-term safety and pharmacokinetics studies that have evaluated individual neutral and acidic cannabinoid blends, but there are no studies in dogs of a combined oral CBDA and CBGA blend for an extended period (11, 20, 26). Chronic dosing is needed to identify any adverse chronic or cumulative effects. Considering CBG has been thought to enhance CBDA absorption when using full spectrum hemp products compared to isolates, pharmacokinetic assessment is essential when examining whole hemp or combination products (30). Since the primary site of cannabinoid metabolism is the liver, and previous studies of oral CBD administration in dogs have shown elevated alkaline phosphatase (ALP) and the potential for alanine aminotransferase (ALT) increases, it is of paramount importance to assess hepatic function (31, 32).

The objective of this study was to examine pharmacokinetics of an equal blend of CBDA and CBGA as major cannabinoids delivered to dogs from a whole hemp extract in a dosing regimen that may lead to therapeutic levels in the serum, and to perform 24-h pharmacokinetics and estimated twice daily dosing steady state concentrations in the serum at 27,57, and 90 days of chronic administration. The second objective was to assess wellness through physical examinations, monthly serum chemistries, and complete blood count evaluations to determine the safety of hemp oil administration. We hypothesized that this would be well tolerated and that CBDA and CBGA would show similar pharmacokinetic profiles with lesser absorption of the minor acidic cannabinoids, THCA and CBCA, found in a whole hemp extract nutraceutical.

## 2 Materials and methods

### 2.1 Animals and treatment

All experimental procedures were approved by the Summit Ridge Farms Institutional Animal Care and Use Committee. Eight adult intact Beagle dogs between 1 and 4 years of age were included in the study. All dogs body condition scores were 5/9 and weighed between 10.15 and 10.95 kilograms at initiation of the study. Male and female sexes were equally represented. Prior to enrollment, all dogs involved in the study were determined to be healthy based on physical examination.

Dogs were individually housed in compliance with the Animal Welfare Act. Temperature was controlled between 10 and 27 °C and a 12-h-light/12-h-dark cycle was maintained. Water was offered *ad libitum.* Dogs were fed twice daily, with approximately 9 h between morning and afternoon feedings. The diet consisted of dry food (Purina Dog Chow [21% protein, 10% fat, 4% crude fiber as fed], Nestle Purina, St. Louis, MO). Daily portions were calculated based on initial body weights and the metabolizable energy of the diet and the daily amount divided equally between feedings of test soft gel administration. After the first week, and every week thereafter, the amount of diet fed was adjusted as needed to maintain weight and body condition.

Dosage of the test article was calculated based on the initial body weight of each dog and remained constant throughout the study. The test article consisted of soft gel capsules containing a full-spectrum hemp extract, primarily containing nearly equal amounts of CBDA and CBGA. The soft gels were available in 2 sizes, one containing 20 mg and one containing 5 mg total cannabinoids. Target dose was 2 mg/kg of CBDA + CBGA. Other minor cannabinoids present in small quantities in the hemp extract were not included in the dosing calculation. Across the group of 8 dogs, the body weights varied very slightly between 10.15 and 10.95 kg and each dog received (1) 20 mg soft gel capsule and (1) 5 mg soft gel capsule at each treatment to achieve the target dose of 2 mg/kg of CBDA (1 mg/kg) + CBGA (1 mg/kg).

Hemp oil soft gels were administered twice daily for 90 days. On Day 1, treatment was administered only once to quantify 24-h single-dose pharmacokinetics of the cannabinoids administered. The Day 0 dose was administered in the morning, immediately followed by ½ the daily ration of food. On Days 2–90, the capsules were given in the morning and 9 h later each afternoon. Each dose was immediately followed by offering ½ the daily food ration. Oral dosing was accomplished by tilting the dog’s head slightly back, gently opening the mouth, quickly placing the soft gels on the back of the tongue, and closing the mouth while keeping the head tilted back for several seconds.

### 2.2 Hemp product quality control assessment

The hemp extract utilized was solubilized in pharmaceutical grade medium chain triglyceride oil and was made into a soft gel capsules in two sizes: one containing 20 mg total cannabinoids, and one containing 5 mg (Ellevet Sciences; Portland, ME). Products were sent for third party analysis at a 17,025 validated Cannabis testing laboratory as part of quality control (Proverde Labs, Milford, MA).

### 2.3 Safety monitoring, physical examination, complete blood count and serum chemistry

During the treatment complete blood count and serum chemistries were assessed at day 0, day 27, day 57, and day 90. (Antech Diagnostic Services, Fountain Valley, CA). The complete blood count parameters included hematocrit (HCT), hemoglobin (Hgb), red blood cell count (RBC), mean corpuscular volume (MCV), mean corpuscular hemoglobin (MCH), mean corpuscular hemoglobin concentration (MCHC), white blood cell count (WBC), segmented neutrophils, lymphocytes, monocytes, eosinophils, basophils and platelets. For serum chemistry, parameters included sodium, potassium, chloride, urea nitrogen, creatinine, calcium, phosphate, magnesium, total protein, albumin, globulin, glucose, alanine aminotransferase (ALT), aspartate aminotransferase (AST), alkaline phosphatase (ALP), gamma-glutamyl transferase (GGT), total bilirubin, triglyceride, cholesterol, and creatine kinase. At each time point above a physical examination including neurological and orthopedic assessment was performed. Throughout the entire 90 day period body weight was collected at 2-week intervals and feed was adjusted to maintain dogs at a body condition score of 5 out of 9. Records of appetite and gastrointestinal disturbances were recorded daily by animal caretakers each morning to track adverse events related to dosing.

### 2.4 Serum cannabinoid analysis

Analysis was performed with a fit-for-purpose liquid chromatography-mass spectrometry (LC/MS/MS) method for measurement of nine cannabinoids and four of their metabolites (ElleVet Sciences, Portland, Maine) (33). Matrix matched calibration curves were generated for each compound using certified reference standards (Cerilliant Corporation, Round Rock, TX) (33). An internal standard solution was generated in methanol at 1 ug/mL containing 7-OH-CBD-d3, 7-COOH-CBD-d3 11-OH-D9-THC-d3, CBDA-d3, CBD-d3, CBGA-d3, CBG-d3, CBC-d3, CBCA-d3, D9-THC-d3, and THCA-A-d3. Dog serum samples were prepared for analysis by adding 100 μL of serum to 250 μL of acetonitrile and 50 μL of internal standard solution. Samples were vortexed for at least 1 min and centrifuged at 10,000 rpm (9,300 g) at 4 °C for 30 min. The supernatant was transferred to autosampler vials for analysis via LC–MS/MS (Agilent 1,260 Infinity II HPLC coupled to an Agilent 6,490 Triple Quadrupole). 5 μL of each sample was injected onto an Restek C18 column (Raptor ARC-18 2.7 μm 150 × 2.1 mm). The column was equilibrated with 50% mobile phase A (0.1% formic acid in water) and 50% mobile phase B (0.1% formic acid in acetonitrile). The column compartment was held at 35 °C. The compounds were eluted by a starting gradient of 50% B held for 0.5 min, ramped to 95% B (0.5–6.0 min), ramped to 100% B (6.0–8.0 min), and held at 100% B (8.0–10.0 min). The column was re-equilibrated to the initial mobile phase composition for 4.0 min. The flow rate was 0.4 mL/min for the entire analysis. The compounds were detected in electrospray ionization positive and/or negative mode as described in Supplementary Table 1. The gas temperature and sheath gas temperature was set to 120 °C and 325 °C, respectively. The gas flow and sheath gas flow was set to 20 L/min and 12 L/min, respectively. The nebulizer was set to 50 psi. The capillary and nozzle voltage was set to 3,500 V and 1,000 V, respectively. Concentrations were calculated using Agilent MassHunter Quantitative Analysis version 10.0 using a linear regression with 1/c weighing based on relative response for each compound.

### 2.5 Pharmacokinetics and statistical analysis

After evaluation of the time dependent cannabinoid and metabolite serum concentrations those found to be above the lower limit of quantification were evaluated using a 24-h non-compartmental pharmacokinetic analysis for each hemp-derived cannabinoid (CBDA, CBGA, THCA, CBCA) utilizing a commercial software system (PK solutions 2.0, Summit PK, Montrose, CO). Semi-log plots were utilized to determine linearity of the elimination profiles. The results generated were maximum serum concentration (Cmax), time to maximal concentrations (Tmax), elimination half-life (T ½), area under the curve to the last time-point (AUC_0-24_), and mean residence time (MRT). Additionally, calculations for Cmax and AUC _0–24_ were divided by dose to provide comparative information on relative absorption and retention of all acidic cannabinoids. All results are presented as the geometric mean, median and range for all results. The program predicts steady state average serum concentrations (Css Ave) based on the assumption that steady state levels are achieved after 5 half-lives with selected frequencies of administration. All cannabinoids and metabolites were examined at days 27, 57 and 90, to represent relative steady state serum concentrations 6 h after the morning dose and reported as means and standard deviations. Serum concentrations of the midpoint-dosing interval across timepoints were compared to the predicted Css average using a Friedman or analysis of variance testing depending on normality (Shapiro Wilk’s testing) with a significant difference set at a *p*-value of 0.05. Results of the body weights, monthly temperatures, heart rates, complete blood counts and serum chemistry parameters were assessed for normality using a Shapiro Wilk’s test. Normally distributed data are reported as means and standard deviations and were tested over time utilizing a one-way analysis of variance and Tukey’s *post-hoc* testing. Non-normally distributed data are presented as median and ranges and were analyzed via Friedman testing with post hoc Dunn’s testing to determine differences between time point 0 and all other timepoints. All *p* values≤ 0.05 were considered significant. All statistical testing and graphing was performed with GraphPad Prism 10.0 (GraphPad Software Inc., LaJolla, CA). Any result that was below the quantifiable limit for the respective cannabinoid was considered 0 for all representation of data.

## 3 Results

### 3.1 External certified 17,025 cannabinoid and quality control analyses

Third party cannabinoid analysis of the soft gels revealed that the cannabinoid profile of the 20 mg soft gels provided 9.0 mg CBDA, 8.0 mg CBGA, 0.4 mg THCA, 0.7 mg CBCA, 0.8 mg CBD, 0.9 mg CBG, 0.1 mg THC, 0.2 mg cannabichromene (CBC) and 0.1 mg cannabinol (CBN). The 5 mg soft gels were comparable in the ratios of cannabinoids at one quarter of the 20 mg dose. All other cannabinoids were below the detectable limit. Third party analysis of the oil also passed for oral consumption for heavy metals, mycotoxins, pesticides, microbials and extraction solvents.

### 3.2 Physical examination and adverse events

During the 3-month trial dogs were observed to eat their daily ration consistently in the morning and evening after the oral dosing each day with no food refusals observed. Vomiting was observed once in 5 dogs, on 5 separate occasions throughout the study (Days 3,18, 25, 37 and 74). Loose stools were observed in 5 dogs on different occasions with 1 dog on day 18, another dog on day 0 and day 1, another on day 57 and day 66, another on day 58 and the last dog on days 43 and 73. This rate of gastrointestinal adverse events was deemed to be within the normal ranges for this colony of beagles. Rectal temperatures monthly were within normal ranges at each time point. Heart rates showed no significant differences over time averaging 113 ± 15 bpm at time 0, 123 ± 14 bpm at day 27, 111 ± 13 bpm at day 57and 118 ± 8 bpm at day 90. Over the course of the 3-month trial the initial mean weight body weight was 10.3 ± 0.3 kg and increased to 10.7 ± 0.4 kg at day 27, and at day 57 to 10.8 ± 0.5 kg. By day 90 the dog’s average weight was 11.0 ± 0.3 kg which was significantly different from the initial body weight (*p* = 0.01).

### 3.3 Complete blood counts

Assessment of complete blood counts over the 90 day period revealed that there were no significant changes over the 3 months for WBC, RBC, hemoglobin, hematocrit, MCHC, platelets, neutrophils, lymphocytes or eosinophils. (Table 1) MCV showed a median increase at day 57 compared to day 90 only (*p* = 0.007). Monocytes showed a similar median increase at day 57 when compared to the initial blood draw median (*p* = 0.04). Although median platelet counts and median MCHC concentrations showed significant differences over time, Dunn’s *post-hoc* analysis could not detect differences between groups. All values were within the reference ranges for all parameters suggesting normal variation rather than treatment effects.

**Table 1:** Mean and standard deviation or median and range for complete blood counts for all dogs (*n* = 8) prior to treatment and days 27, 57 and 90.

Parameter (ref range)	Baseline	Day 27	Day 57	Day 90	*p*-value
WBC (4.0–15.5 × 10^3/uL)	7.4 + 1.6	6.4 ± 1.0	6.1 ± 1.4	6.1 + 0.9	0.34
RBC (4.8–9.3 × 106^6/uL)	7.2 ± 0.3	7.5 ± 0.6	7.5 ± 0.6	7.5 ± 0.6	0.21
Hgb (12.1–20.3 g/dL)	17.3 (15.4–18.7)	18.5 (14.1–19.5)	18.2 (15.2–19.6)	17.7 (15.5–19.4)	0.15
HCT (36–60%)	55 + 3	56 ± 5	55 ± 4	57 ± 5	0.39
MCV (60–77 fL)	77 + 3	75 ± 2	73 ± 2	76 ± 2*	0.03
MCH (19.5-30.0 pg)	23.8 + 0.7	23.8 ± 0.9	23.9 ± 1.2	23.5 ± 1.0	0.66
MCHC (32–36%)	31.0 (30.0–32.0)	32.5 (30–33)	33.0 (29–34)	31.5 (29–32)	0.02
Platelets (170–400 × 10^3/uL)	283 + 162	325 ± 111	287 ± 79	248 ± 53	0.02
Neutrophils (2060–10,600/μL)	4,679 + 1,650	4,117 ± 860	4,050 ± 1,214	4,117 ± 860	0.13
Lymphocytes (690–4,500/μL)	1,615 + 343	1,632 ± 486	1,568 ± 361	1,632 ± 486	0.16
Monocytes (0–840/μL)	339 (215–532)	273 (174–550)	248 (163–365)**	303 (186–434)	0.05
Eosinophils (0–1,200/μL)	325 (200–1,078)	220 (189–496)	208 (165–594)	277 (192–335)	0.17

*indicates significance between day 57 and day 90. **indicates significance between baseline and day 57.

### 3.4 Serum biochemistry analytes

Assessment of the serum chemistry screens over the duration of the trial showed no significant differences in TP, albumin, globulin, AST, ALT, ALP, GGT, total bilirubin, phosphorus, magnesium, potassium, sodium, cholesterol, triglyceride or CK.(Table 2) One way analysis of variance revealed a significant difference over time for BUN, however Tukey’s *post hoc* analysis showed no differences between the individual time points. Serum creatinine was increased from the initial baseline time point at day 57 (*p* = 0.02) and day 90(*p* = 0.004) time points, yet no values were above or below reference ranges. Similarly, serum calcium was elevated compared to pre-treatment at both day 27(*p* = 0.003) and day 57 time point (*p* = 0.006), with no significant differences noted at 90 days. Serum glucose decreased at day 90 compared to day 27 (*p* = 0.02) and day 57 (*p* = 0.02). Serum chloride concentrations were significantly increased at day 27 compared to day 57 (*p* = 0.002) and day 90 (*p* = 0.005). For all serum electrolytes and glucose the values never fell outside of the established reference ranges and are assumed to be normal variation.

**Table 2:** Mean and standard deviation or median and range for serum chemistry for all dogs (*n* = 8) prior to treatment and again on days 27, 57, and 90.

Parameter (ref range)	Baseline	Day 27	Day 57	Day 90	*p*-value
TP (5.0–7.4 g/dL)	5.7 ± 0.5	5.6 ± 0.8	5.9 ± 0.5	5.58 ± 0.34	0.18
Albumin (2.7–4.4 g/dL)	3.3 ± 0.3	3.4 ± 0.4	3.4 ± 0.3	3.33 ± 0.31	0.11
Globulin (1.6–3.6 g/dL)	2.5 ± 0.4	2.4 ± 0.4	2.5 ± 0.4	2.3 ± 0.4	0.21
AST (15–66 U/L)	24 ± 5	25 ± 5	23 ± 4	21 ± 3	0.58
ALT (12–118 U/L)	34 ± 12	29 ± 10	28 ± 8	29 ± 7	0.09
ALP (5–131 U/L)	42 ± 18	43 ± 13	35 ± 13	35 ± 10	0.65
GGT (1–12 U/L)	4 ± 1	4 ± 1	4 ± 2	4 ± 1	0.24
Total Bilirubin (0.3–0.4 mg/dL)	0.1 (0.1–0.1)	0.1 (0.1–0.2)	0.1 (0.1–0.2)	0.1 (0.1–0.2)	0.49
Blood Urea Nitrogen (6–31 mg/dL)	12 ± 2	14 ± 3	14 ± 2	14 ± 3	0.02
Creatinine (0.5–1.6 mg/dL)	0.5 ± 0.1	0.5 ± 0.1	0.6 ± 0.1*	0.6 ± 0.1*	< 0.001
Phosphorous (2.5–6.0 mg/dL)	4.2 ± 0.6	4.3 ± 0.5	3.9 ± 0.3	4.1 ± 0.3	0.11
Glucose (70–138 mg/dL)	84 ± 6	87 ± 8	87 ± 6	81 ± 5**	0.00
Calcium (8.9–11.4 mg/dL)	9.8 (9.1–9.8)	10.2 (9.6–10.5)*	10.3 (9.5–10.4)*	9.8 (9.5–10.0)	< 0.001
Magnesium (1.5–2.5 mEq/L)	1.5 (1.3–1.6)	1.5 (1.4–1.6)	1.6 (1.4–1.6)	1.5 (1.4–1.6)	0.27
Sodium (139–154 mEq/L)	149 (142–149)	147 (146–152)	147 (145–148)	147 (145–149)	0.55
Potassium (3.6–5.5 mEq/L)	4.5 (4.2–5.3)	4.6 (4.3–5.2)	4.5 (4.3–5.0)	4.6 (4.3–5.0)	0.46
Chloride (102–120 mEq/L)	113 (109–114)	115 (112–117)***	111 (109–112)	112 (111–114)	0.02
Cholesterol (92–324 mg/dL)	137 ± 39	153 ± 34	144 ± 30	137 ± 25	0.23
Triglycerides (29–291 mg/dL)	61 ± 23	50 ± 11	57 ± 19	63 ± 14	0.20
Creatine Kinase (59–895 U/L)	78 ± 17	74 ± 13	73 ± 14	63 ± 14	0.34

*indicates significant difference from baseline. **indicates significant difference between day 27 and day 57 compared to day 90. ***indicates significant difference between day 57 and day 90 compared to day 27.

### 3.5 Pharmacokinetics

Of the 13 cannabinoids and metabolites assessed in the serum only 4 acidic cannabinoids could be reliably measured above limit of quantitation in the serum of dogs during the 24-h pharmacokinetic assessment (Table 3), or the monthly serum draws at day 27, 57 and 90, 6 h after a typical morning treatment with the test cannabinoid soft gel capsules. The pharmacokinetics reveal a similar CBDA and CBGA Tmax, and MRT, with a slightly higher Cmax and AUC_0-24_ for CBDA than CBGA. THCA and CBCA show similar Tmax with slightly longer MRT and T1/2 elimination than the more prominent cannabinoid acids in the formulation, with Cmax and AUC being lower due to the lower dosing. Cmax and AUC of CBDA and CBGA showed similar Cmax and AUC based on dose consumed, while CBCA and THCA show far higher Cmax and AUC based on dose consumed in the 24-h pharmacokinetic screen.

**Table 3:** Geometric mean, median and range for 24 h pharmacokinetic non-compartmental modeling of CBDA, CBGA, THCA and CBCA displaying Cmax, T max, T1/2 life, AUC, MRT and Cmax and AUC divided by dose (*n* = 8).

Cannabinoid	Cmax (ng/mL)	Tmax (h)	T1/2 el (h)	AUC (0–24 ng-h/mL)	MRT	Cmax/Dose	AUC/Dose
CBDA	621; 576 (181–1,268)	1.2; 1 (1–4)	2.7; 3.0 (1.7–3.7)	1,383; 1,185 (949–2,519)	3.3; 3.1 (2.3–5.0)	572; 537 (174–1,153)	1,255; 1,087 (857–2,354)
CBGA	428; 478 (116–876)	1.2; 1 (1–4)	1.9; 1.8 (1.7–2.4)	930; 896 (612–1,359)	2.9; 2.7 (2.3–4.6)	454; 498 (128–913)	987; 964 (672–1,430)
THCA	66; 68 (38–118)	1.2; 1 (1–4)	4.4; 4.5 (3.2–5.6)	282; 279 (228–359)	5.3; 5 (4.9–9.2)	1,344; 1,373 (762–2,376)	5,641; 5,574 (4,568-7,174)
CBCA	145; 148 (74–466)	1.2; 1 (1–4)	3.4; 3.2 (3.1–4.2)	641; 613 (571–805)	4.7; 4.3 (4.0–7.0)	1,605; 1,641 (818–2,893)	7,126; 6,807 (6,347–8,940)

For the monthly blood draws the predicted steady state based on pharmacokinetic modeling showed no significant differences across all the acidic cannabinoids by 90 days, regardless of the lower geometric means and medians due to the large ranges observed. (Table 4) At day 27 all acidic cannabinoids were significantly lower than the predicted steady state except for THCA and at day 57 only CBGA was significantly lower than the predicted twice daily dosing steady state concentrations.

**Table 4:** Geometric mean, median and range of monthly steady state pharmacokinetics compared to the predicted steady state concentration from initial 24-h pharmacokinetic modeling based on twice daily dosing (*n* = 8).

Cannabinoid	Ave Pred. SS ng/mL	Day 27 ng/mL	Day 57 ng/mL	Day 90 ng/mL
CBDA	115; 100 (79–173)	24; 30 (25–101)*	47; 55 (9–99)	59; 60 (24–102)
CBGA	76; 75 (51–98)	27; 24 (19–67)*	25; 26 (5–63)*	25; 40 (10–69)
THCA	24; 24 (19–31)	16; 14 (11–30)	15; 15 (4–25)	18; 19 (13–25)
CBCA	55; 54 (50–67)	37; 36 (26–67)*	32; 38 (7–66)	42: 43 (26–62)

*indicates a statistically significant difference from the predicted steady state at 6 h post morning dosing at monthly intervals (*p* < 0.05).

## 4 Discussion

Over the course of 90 days, this study assessed the safety and pharmacokinetic profile of whole hemp extracts, which included equal amounts of CBDA and CBGA, in healthy adult Beagles. The findings indicate that the formulation is generally well-tolerated and that acidic cannabinoids show no alteration in serum biochemical parameters and higher bioavailability than neutral cannabinoids, indicating the potential for further efficacy research. Creatinine was the only chemistry parameter that showed a mild rise over the study time but remained well within reference ranges for all dogs. It is also noted that the dogs being fed twice a day versus typical once a day feeding in the colony resulted in approximately one half of a kilogram of weight gain on average, which might be due to an increase in muscle mass leading to the mild increase in creatinine (34). Other serum and complete blood count alterations appeared to be spurious at variable time points, are very mild and within reference ranges, and likely related to biological variation. For example, serum calcium rose from the baseline at 28 days and 57 days but was similar to pretreatment by day 90. Although hypocalcemia has been noted in horses in one study with CBD treatment at 1.5 and 3 mg per kg, all the dogs remained well within reference ranges (35).

No significant changes were noted in the complete blood counts or in most of the serum chemistries, including liver enzymes, which aligns with the chronic administration being well-tolerated. This outcome favorably compares to some other CBD safety studies in dogs, where CBD administration has been associated with an increase in ALP without a corresponding increase in ALT (31, 32). The lack of an increase in ALP in the current study for the full spectrum hemp extract suggests a lesser impact on the liver compared to CBD isolates at similar dosing to this extract, or a difference in healthy younger dog treatment versus treatment of dogs with comorbidities (1, 36). CBDA may have increased bioavailability due to its more hydrophilic nature considering the additional carboxyl group or decreased first-pass hepatic metabolism. According to an assessment of metabolism and drug–drug interactions involving canine cytochrome P-450 and UDP-glucuronosyltransferase, CBDA is primarily glucuronidated with minimal modification through the cytochrome P450 (CYP) enzymes, unlike CBD (19). We do not know how CBGA, THCA, and CBCA are metabolized; however, it is likely different from their neutral counterparts which are also sparsely studied.

In previous research, CBDA has consistently demonstrated improved bioavailability and higher plasma concentrations in dogs compared to its decarboxylated equivalent, cannabidiol (CBD) (11–15). This enhanced systemic exposure is hypothesized to be facilitated by reduced metabolism and interactions with drug efflux pumps, such as breast cancer resistance protein (30). However, the overall pharmacokinetic profile for the entire acidic cannabinoid class is highly complex, and acidic cannabinoids are generally considered to be more stable when formulated within a complex full-spectrum extract matrix compared to singular isolated compounds (12, 37). Like CBDA, a recent study in dogs showed measurably superior systemic absorption and retention of CBGA than its neutral equivalent, CBG (29). This is further supported by the composition of the full hemp extract, as CBDA is a well-known constituent of hemp and even hemp seed oil (38) whose absorption dynamics are superior to CBD in an oil-based full-spectrum extract like the one used in this study.

When comparing CBDA and CBGA absorption to other species, studies in cattle (39, 40), goats (41), horses (14, 15, 37), dogs (12, 33), rabbits (15), and humans (13, 42) have similarly reported serum/plasma CBDA concentrations often exceeded CBD when administered via oral hemp products. These studies highlight the dramatically different absorption and retention dynamics of acidic cannabinoids compared to the neutral forms where the improved pharmacokinetics range from 3 to 50 fold different depending on dosing and species. Additionally, the predicted steady state based on twice daily dosing was similar to the actual spot measurements monthly for the acidic cannabinoids suggesting that the non-compartmental analysis was likely appropriate.

Of particular interest in this study is the absorption of THCA and CBCA, showing measurable serum concentrations when compared to their neutral equivalents. Even more surprising was their absorption/retention when compared to CBDA and CBGA when adjusted for the 10-fold decrease in dose. THCA and CBCA had better systemic availability/retention than CBDA and CBGA. CBCA had the largest systemic exposure when normalized by dose, with a maximum concentration of 2,152 ng/mL/mg and an AUC of 9,339 ng-h/mL/mg. THCA was similar, with a maximum concentration of 1,620 ng/mL/mg and an AUC of 7,066 ng-h/mL/mg. Compared to CBDA (AUC dosage of 1,669 ng-h/mL/mg) and CBGA (AUC dose of 1,234 ng-h/mL/mg), these values were substantially higher. This result clearly indicates that other minor acidic cannabinoids, such THCA and CBCA, may exhibit better absorption than CBDA and the neutral forms, potentially increasing systemic exposure on a dose-by-dose basis warranting further study of these minor cannabinoids found in hemp.

CBDA and CBGA, along with other acidic cannabinoids, have been shown to have anti-inflammatory and antioxidant capabilities (43), antimicrobial (44) and antiviral properties (45). CBDA has been identified as a potent anti-emetic compound, demonstrating superior efficacy to CBD in reducing nausea and vomiting in male and female rat models (46). CBDA has demonstrated reduction on thermal pain sensitivity (47), attenuated cognitive impairments and anxiolytic effects in certain rodent models of stress (48, 49). *In vivo* oral dosing studies have found that a CBD/CBDA rich hemp abstract ameliorated Chronic Resistant Stress (CRS) cognitive impairment and reduced specific markers of microglial activation and astrocytic structural protein in rats (48).

CBGA has been linked to neuroprotection in models of Parkinson’s disease (50) and has demonstrated seizure migration in rodent models (51) The anticonvulsant and analgesic properties of acidic cannabinoids may be explained by their ability to inhibit voltage-gated sodium channels (52). Emerging evidence highlights potential organ protection through inhibition of inflammation and concomitant fibrosis in nephropathy (24). One mechanism identified may be through CBGA and other acidic cannabinoids demonstrating the ability to suppress proinflammatory cytokine release by blocking store-operated calcium entry (53). Another study shows CBGA’s potent inhibitory effect on the TRPM7 ion channel through its kinase domain. TRPM7 is implicated in diseases such as cancer, stroke, and kidney disease (54). Both CBDA and CBGA are also known to modulate the cyclooxygenase (COX) pathway, similar to non-steroidal anti-inflammatory drugs (NSAIDs) with a COX-2 preferential activity, while neutral cannabinoids have little to no activity on COX enzymes (22, 26).

Although THCA and CBCA are less well studied, THCA has been shown to have neuroprotective effects in models of neurodegeneration (55, 56) Veterinary studies include *in vivo* treatment of steers with full spectrum hemp to provide CBDA at 5.5 mg/kg daily as the predominant cannabinoid with lower concentrations of CBGA, THCA, and CBCA Physiologically this acidic cannabinoid treatment was shown to improve activity and to reduce biomarkers of stress and inflammation in steers (40). These diverse mechanisms support the broad therapeutic utility of a whole hemp extract rich in acidic cannabinoids. Although CBDA and CBGA have not been linked to mitigation of stress like behaviors in dogs, CBD and CBD/CBG based products have been used in situational use and chronic anxiety behaviors with mixed results for improving kenneling in shelter dogs and possibly mitigating aggressive behaviors (57–60). Unfortunately, this normal population of beagles showed no gross positive or negative behavioral effects of the treatment over time and were not evaluated by a veterinary behaviorist.

The primary limitations of this study are in its scope of a small sample size, and controlled environment, which may not reflect a heterogeneous large canine population outside of the contract research laboratory environment. This environment led to constraints in dosing frequency being at approximately 7 a.m. and between 3–4 p.m. in the afternoon, which is not prototypical for every 12 h dosing which may have slightly altered the pharmacokinetic profile. Across this small population the ranges in Cmax and AUC for the acidic cannabinoids were 7–8 fold different across the population suggesting wide variability in absorption and/or retention which may not fully represent larger populations of dogs and different breeds. This brings to light that the inclusion of just one canine breed does not account for differences across a diverse canine population including different breeds, ages, weights, concurrent medications or uncontrolled home environments and diets. In addition, our study did not have a control population. This made the bloodwork changes, although all within reference range, somewhat tenuous, as a placebo population was not included. Although a 90 day study supports safety at the specific dose, longer studies at variable dosing would establish definitive no observable adverse effects limits and are warranted moving forward.

## 5 Conclusion

This initial investigation into the safety of a CBDA and CBGA blended whole hemp product for oral consumption in dogs was well tolerated for 90 days of administration with no overt adverse events associated with this specific application. The clinical, hematological, and serum chemistry parameters, including the liver enzymes, suggest a more favorable safety profile compared to isolated CBD in the literature. Absorption of both CBDA and CBGA was confirmed by pharmacokinetic evaluation, consistent with previous studies suggesting higher systemic availability for acidic cannabinoids over their neutral counterparts. This study also showed an unexpectedly high systemic availability for the minor acidic cannabinoids CBCA and THCA. While the monthly observed steady-state concentrations for the major acidic cannabinoids were slightly lower than predicted from initial 24-h pharmacokinetic modeling, they were often not significantly different, suggesting the modeling was effective. The lack of adverse effects, coupled with the known superior bioavailability and anti-inflammatory/antioxidant potential of acidic cannabinoids, and the high absorption of CBCA and THCA, supports further investigation into therapeutic uses.

## Data Availability

The raw data supporting the conclusions of this article will be made available by the authors, without undue reservation.
