# Supplementary material for: Physiological effect and pharmacokinetic evaluation of combined oral administration of cannabidiolic acid and cannabigerolic acid in dogs
Source: Front Vet Sci. 2026 Feb 25;13:1779760. doi: 10.3389/fvets.2026.1779760 (PMC12975459; doi:10.3389/fvets.2026.1779760)
Supplement: Supplementary file 1 [file Data_Sheet_1.PDF]

Supplementary Table 1: LC-MS/MS parameters and calibration curve range of lower (LLOQ) and upper limit of quantitation (ULOQ) in dog serum.

**Compound name**	**Precursor Ion**	**Product Ion**	**Polarity**	**Range Cal Curve LLOQ/ULOQ (ng/mL)**
11-OH-D9-THC	331.47	313.8	Positive	1-1,500
11-OH-D9-THC	331.47	193.3	Positive	
11-OH-D9-THC	331.47	122.9	Positive	
6-OH-CBD	329.45	311.1	Negative	1-1,500
6-OH-CBD	329.45	173.1	Negative	
6-OH-CBD	329.45	157.8	Negative	
7-COOH-CBD	343.43	299.1	Negative	1-1,500
7-COOH-CBD	343.43	297	Negative	
7-COOH-CBD	343.43	231	Negative	
7-OH-CBD	329.45	311.1	Negative	1-1,500
7-OH-CBD	329.45	261.1	Negative	
7-OH-CBD	329.45	178.9	Negative	
CBC	315.47	193.4	Positive	1-1,500
CBC	315.47	123.1	Positive	
CBC	315.47	93.1	Positive	
CBCA	357.46	339.1	Negative	1-1,500
CBCA	357.46	313.1	Negative	
CBCA	357.46	191	Negative	
CBD	315.47	193.4	Positive	1-1,500
CBD	315.47	123.3	Positive	
CBD	315.47	93	Positive	
CBDA	359.48	341.3	Positive	1-1,500
CBDA	359.48	261.5	Positive	
CBDA	359.48	219.4	Positive	
CBG	317.5	193.3	Positive	1-1,500
CBG	317.5	123.1	Positive	
CBG	317.5	95.2	Positive	
CBGA	361.5	219.4	Positive	1-1,500
CBGA	361.5	149.1	Positive	
CBGA	361.5	135.1	Positive	
CBT	315.5	123.3	Positive	1-1,500
CBT	315.5	193.4	Positive	
D3-11-OH-D9-THC	334.49	316.4	Positive	
D3-11-OH-D9-THC	334.49	196.3	Positive	
D3-11-OH-D9-THC	334.49	105.3	Positive	

D3-7-OH-CBD	332.7	172.9	Negative	
D3-7-OH-CBD	332.47	314.2	Negative	
D3-7-OH-CBD	332.47	264.1	Negative	
D3-7-COOH-CBD	346.5	302.2	Negative	
D3-7-COOH-CBD	346.5	300.1	Negative	
D3-7-COOH-CBD	346.5	231.0	Negative	
D3-CBC	318.5	196.5	Positive	
D3-CBC	318.5	123.1	Positive	
D3-CBC	318.5	93.1	Positive	
D3-CBCA	360.5	194.0	Negative	
D3-CBCA	360.5	316.1	Negative	
D3-CBD	318.49	196.3	Positive	
D3-CBD	318.49	123.2	Positive	
D3-CBD	318.49	93.1	Positive	
D3-CBDA	362.5	91.1	Positive	
D3-CBDA	362.5	344.8	Positive	
D3-CBDA	362.5	222.5	Positive	
D3-CBG	320.5	196.4	Positive	
D3-CBG	320.5	123.3	Positive	
D3-CBG	320.5	95.2	Positive	
D3-CBGA	364.5	222.4	Positive	
D3-CBGA	364.5	149.1	Positive	
D3-CBGA	364.5	135.1	Positive	
D3-D9-THC	318.49	196.4	Positive	
D3-D9-THC	318.49	123.2	Positive	
D3-D9-THC	318.49	93	Positive	
D3-THCA	360.49	316.1	Negative	
D3-THCA	360.49	248	Negative	
D3-THCA	360.49	194	Negative	
D9-THC	315.47	193.4	Positive	
D9-THC	315.47	123.1	Positive	
D9-THC	315.47	93	Positive	
THCA	357.46	313.1	Negative	1-1,500
THCA	357.46	245	Negative	
THCA	357.46	191	Negative	
